# The application of fecal microbiota transplantation in Parkinson’s disease

**DOI:** 10.3389/fnagi.2025.1713899

**Published:** 2025-12-17

**Authors:** Boyan Wang, Di Ma, Na Li, Tianfei Luo

**Affiliations:** Department of Neurology, First Hospital of Jilin University, Changchu, China

**Keywords:** fecal microbiota transplantation, gut microbiota, microbiota-gut-brain axis, Parkinson’s disease, oxidative stress

## Abstract

**Background:**

Parkinson’s disease (PD) is a multisystem neurodegenerative disorder characterized by the aggregation of *α*-synuclein (α-syn) in dopaminergic neurons of the substantia nigra. The pathogenesis of PD remains incompletely understood, and disease-modifying therapies are lacking. Emerging evidence suggests that gut microbiota and their metabolites influence both intestinal and central nervous system (CNS) functions via the microbiota-gut-brain axis (MGBA). Recent studies have identified dysbiosis in the gut microbiota of PD patients, which may contribute to disease progression through two primary mechanisms: First, increased intestinal permeability, allowing pro-inflammatory factors and microbial metabolites to affect the enteric nervous system (ENS) and subsequently spread to the CNS via the vagal neurons; Secondly, disruption of the Blood–Brain barrier (BBB), leading to neuroinflammation and aberrant *α*-syn aggregation, ultimately resulting in dopaminergic neuron degeneration. These findings underscore the critical role of the MGBA in PD pathogenesis, which makes gut microbiota modulation a promising therapeutic target.

**Highlights:**

This review synthesizes current knowledge on gut microbiota alterations in PD and evaluates the potential of fecal microbiota transplantation (FMT) as an adjunctive therapy to alleviate motor and non-motor symptoms and slow disease progression.

**Conclusion:**

FMT has demonstrated efficacy in ameliorating PD symptoms via the MGBA. However, further preclinical and clinical studies are needed to fully elucidate its mechanisms and optimize therapeutic protocols. Targeting the gut microbiota may offer novel biomarkers and intervention strategies for PD.

## 1 Introduction

Parkinson’s disease (PD) is a prevalent neurodegenerative disorder predominantly affecting middle-aged and elderly populations, with an estimated global prevalence of nearly 10 million cases. As the second most common neurodegenerative disease after Alzheimer’s disease, PD is pathologically characterized by two hallmark features: First, progressive degeneration of dopaminergic neurons in the substantia nigra pars compacta; Secondly, the formation of Lewy bodies and Lewy neurites composed primarily of aggregated *α*-syn (Postuma et al., 2015). Lewy bodies, the pathological hallmark of PD, are primarily composed of misfolded*α*-syn, ubiquitin, and heat shock proteins. Notably, the aberrant aggregation of α-syn—encoded by the SNCA gene—is recognized as a central driver of progressive neurodegeneration in PD (Jucker and Walker, 2018; Mehra et al., 2019). Moreover, the pathogenesis of PD involves multiple interconnected mechanisms, including genetic predisposition, *α*-syn aggregation, impaired protein clearance, mitochondrial dysfunction, ferroptosis, neuroinflammation, and oxidative stress (Wang Z. L. et al., 2022; Yildirim et al., 2022). The multifaceted and incompletely elucidated pathogenesis of PD has hindered the development of disease-modifying therapies. PD patients manifest a complex array of clinical features encompassing both motor symptoms, including resting tremor, rigidity, bradykinesia, and postural instability, and non-motor symptoms, such as hyposmia, sleep disturbances, cognitive impairment, autonomic dysfunction, and gastrointestinal disorders (Poewe et al., 2017). Constipation represents one of the most prevalent non-motor symptoms in PD, frequently manifesting prior to the onset of characteristic motor impairments. Emerging evidence from advanced biotechnological research has elucidated a bidirectional communication pathway between gut microbiota and the CNS, termed the MGBA (Martin et al., 2018; You et al., 2024). MGBA comprises four principal components: CNS, ENS, autonomic nervous system (ANS), and Hypothalamic–Pituitary–Adrenal (HPA) axis. Clinical investigations have demonstrated that the MGBA plays a pivotal role not only in maintaining gastrointestinal homeostasis but also in the pathogenesis of PD. The gut microbiota primarily influences these processes through three distinct pathways: Neural signaling, Immune modulation (Reigstad et al., 2014), and Endocrine regulation. These three pathways function both independently and synergistically to maintain gut microbial homeostasis.

Accumulating evidence indicates that gut dysbiosis in PD patients and animal models contributes to disease progression and pathogenesis through multiple mechanisms, including: increasing intestinal permeability, exacerbatting neuroinflammation, and accumulation of pathological *α*-syn fibrils (Sampson et al., 2016). These findings underscore the pivotal role of the microbiota-gut-brain axis (MGBA) in the pathogenesis of Parkinson’s disease (PD). Current methodological approaches for investigating MGBA interactions can be categorized into two principal strategies: Utilization of germ-free murine models coupled with FMT, wherein gut microbiota from healthy donors are transplanted into recipients to elucidate the regulatory effects of either intact microbial communities or specific bacterial taxa on MGBA function (Fang, 2019); Administering probiotic interventions to established murine models exhibiting either gut dysbiosis or neurobehavioral abnormalities. This strategy enables systematic evaluation of symptom amelioration, thereby functionally validating microbiota-mediated modulation of the MGBA (Hsieh et al., 2020). This review primarily focuses on the therapeutic applications of FMT in PD, aiming to establish a theoretical framework for targeting gut microbiota in PD prevention and treatment. A comprehensive critical analysis is presented herein.

## 2 The relationship between the MGBA and PD

### 2.1 Gut microbiota involvement in PD pathogenesis

PD is a neurodegenerative disorder predominantly affecting middle-aged and elderly populations, clinically characterized by cardinal motor manifestations including resting tremor, rigidity, bradykinesia, and postural instability. Substantial evidence indicates that non-motor symptoms, particularly gastrointestinal disturbances, affective disorders, sleep abnormalities, sensory deficits, cognitive impairment, and autonomic dysfunction—frequently precede motor manifestations by several years and demonstrate progressive exacerbation with disease duration.

Among these non-motor manifestations, gastrointestinal dysfunction represents the most prevalent symptom complex in PD patients. The human gastrointestinal tract harbors a diverse microbial ecosystem that bidirectionally modulates both motility and endocrine functions. This intestinal microbiota comprises a dynamic consortium of microorganisms, including bacteria, archaea, viruses, and eukaryotic organisms (e.g., fungi and protozoa) that occupying distinct ecological niches throughout the gastrointestinal lumen, collectively termed the gut microbiome (Arumugam et al., 2011). The metabolic activities and complex interactions of gut microbiota significantly influence host physiological homeostasis and disease susceptibility. As the “second genome” of the human body, the gut microbiome plays a fundamental role in both the initiation and progression of neurodegenerative disorders (Zhu et al., 2010). The commensal gut microbiota maintains intestinal homeostasis through three principal mechanisms: biosynthesis of essential micronutrients,including vitamins, amino acids, and lipids; modulation of neural signaling pathways; and regulation of immune-endocrine crosstalk. These coordinated functions collectively sustain the MGBA and preserve intestinal microenvironmental stability (Ma et al., 2019; Cook and Mansuy-Aubert, 2022). In recent years, the gut microbiome has emerged as a focal point in PD research, with accumulating evidence implicating its involvement in multiple facets of PD pathogenesis (Scheperjans et al., 2015), clinical phenotype (Vascellari et al., 2020), therapeutic effect of levodopa (Beckers et al., 2022) and relevant effects of disease-modification (Chen and Lin, 2022; Lin et al., 2023). A landmark study recently published in Nat Microbiol (Procházková et al., 2024) has established that intra-individual variations in gut microbiome homeostasis and metabolic activity significantly correlate with alterations in fecal water content and pH levels. Non-motor symptoms of PD are predominantly characterized by gastrointestinal dysfunction and may precede motor symptoms by many years.

Braak and colleagues postulated that *α*-syn pathology originates in the dorsal motor nucleus of the vagus nerve (DMNV) and the anterior olfactory nucleus. Through systematic postmortem neuropathological examinations of PD patients, they advanced the “gut-origin hypothesis” of PD pathogenesis (Klingelhoefer and Reichmann, 2015; Breen et al., 2019). This hypothesis proposes that pathological *α*-syn initially aggregates in the ENS and subsequently undergoes retrograde axonal transport via vagal nerve fibers to the substantia nigra and cerebral cortex. The hallmark neuropathological feature of PD involves the formation of Lewy bodies—intraneuronal cytoplasmic inclusions composed of misfolded *α*-syn aggregates. Current evidence suggests that these aberrant α-syn assemblies contribute to neurodegeneration in both PD patients and animal models through mitochondrial dysfunction-mediated pathways (Sato et al., 2013). The aggregated *α*-syn form fibrillar structures that propagate throughout distinct brain regions in a prion-like manner, exhibiting self-templating properties and cell-to-cell transmission characteristics (Peng et al., 2020). Trancikova et al. (2012) have proposed that mitochondrial dysfunction and oxidative stress associated with Parkinson’s disease (PD) may contribute to pathological *α*-syn aggregation. Notably, the misfolding of α-syn has been identified as a primary causative factor underlying the degeneration of dopaminergic neurons in the substantia nigra of PD patients (Odaka et al., 2003). Accumulating evidence suggests that prodromal features of Parkinson’s disease (PD), including constipation and *α*-syn pathology, can be detected several years prior to clinical diagnosis, potentially serving as early biomarkers for PD risk stratification (Pfeiffer, 2016). However, the precise timing of α-syn entry into the ENS relative to the onset of motor symptoms remains experimentally undetermined. Based on existing evidence, including the “Braak hypothesis” (Braak et al., 2006; Hawkes et al., 2010), we reasonably estimate that pathological *α*-syn likely infiltrates the ENS 10 to 20 years before motor manifestations of PD. Mounting experimental and neuropathological evidence demonstrates that *α*-syn pathology originates preferentially in the ENS, providing compelling support for the gut-to-brain propagation hypothesis via vagal nerve pathways in PD pathogenesis (Wang J. et al., 2022). A prospective study (Abbott et al., 2001), the Honolulu Heart Program (HHP), investigated bowel habits in 6,790 elderly men without PD. Participants were monitored for 24 years, during which 96 individuals developed PD after an average latency of 10 years (range: 5 months to 19 years). Subsequent autopsies of deceased PD patients and controls revealed phosphorylated *α*-syn deposits in both the central and enteric nervous systems during early disease stages, with distribution patterns closely aligning with Braak staging. Intriguingly, injection of pathological α-syn from PD patients into the gut of rodents demonstrated its transmission to the brain via the vagus nerve, ultimately leading to substantia nigra neuronal loss and motor deficits. We posit that presence does not equate to pathogenicity; while α-syn pathology in the enteric nervous system may represent an initial event, progression to clinically diagnosable disease requires prolonged accumulation and propagation. This emerging frontier necessitates further rigorous clinical investigation for validation.

Tang et al. (2013) provided mechanistic evidence that *α*-syn undergoes bidirectional transport across the BBB. Their findings further suggest that lipopolysaccharide (LPS) challenge may enhance α-syn brain uptake by compromising BBB integrity. In murine models, LPS exposure was shown to induce mitochondrial dysfunction through microglial NADPH oxidase activation, thereby initiating neurotoxic cascades (Hu and Wang, 2016). Consequently, LPS has been widely adopted in murine models to recapitulate Parkinsonian pathology. Emerging evidence also highlights an intriguing association between gut-derived proteins and cognitive function. Notably, bacterial production of amyloid proteins has been experimentally demonstrated to exacerbate α-syn pathology in aged rodent models.

Rotenone, a naturally occurring pesticide with high lipophilicity, readily crosses both the BBB and cellular membranes. These properties have established its widespread utility in generating experimental models of PD (Ibarra-Gutiérrez et al., 2023). Rotenone serves as a potent and selective high-affinity inhibitor of mitochondrial complex I (NADH: ubiquinone oxidoreductase) in the electron transport chain (Hineno et al., 2011), Inhibition of mitochondrial complex I activity induces a cascade of pathological events: Elevated reactive oxygen species (ROS) production; Impaired adenosine triphosphate (ATP) synthesis; Mitochondrial membrane depolarization. These alterations disrupt electron transfer from NADH to coenzyme Q, ultimately triggering apoptotic neuronal death (Ratan et al., 2024). Rotenone-induced PD models faithfully recapitulate both the behavioral phenotypes and neuropathological hallmarks observed in clinical PD. Notably, emerging evidence from chronic rotenone exposure models demonstrates that gastrointestinal dysfunction precedes motor impairments, further supporting the gut-origin hypothesis of PD pathogenesis (Bhattarai et al., 2021). Researches have demonstrated that chronic rotenone administration induces tyrosine hydroxylase-positive neuronal loss independent of gut microbiota status, while the subsequent development of motor deficits requires microbial colonization. Furthermore, their work revealed that prolonged rotenone exposure facilitates retrograde *α*-syn propagation to the CNS. Kim et al. (2019) provided experimental evidence that vagotomy effectively blocks the retrograde transport of α-syn fibrils to the brain, thereby preventing associated neurodegeneration and behavioral deficits in their PD model system. A research of professor Jintai Yu (Wu et al., 2025) teamidentified FAM171A2, a neuronal membrane protein, as a critical mediator of pathological α-syn propagation. Building on this discovery, they developed a novel FAM171A2-targeting therapeutic agent that effectively inhibits α-syn transmission throughout all stages of PD progression. Notably, within the context of gut microbiota-PD interactions, murine models of PD exhibit significant alterations in intestinal function that manifest prior to the onset of motor symptoms, further supporting the prodromal gut involvement in disease pathogenesis (Gries et al., 2021). Collectively, these findings provide compelling evidence for a robust association between specific gut microbiota compositions and the pathophysiology of PD.

### 2.2 The pathogenic link between gut microbiota dysbiosis and PD

#### 2.2.1 Gut microbiota dysbiosis

Gut microbiota dysbiosis, defined as structural and/or functional alterations in intestinal microbial communities, has been extensively documented in patients with various neurodegenerative disorders (Rusch et al., 2023). The initial colonization of gut microbiota occurs during the early postnatal period, where microbial transmission from maternal vaginal canal, skin surfaces, and breast milk establishes the neonatal intestinal microbial ecosystem (Ma et al., 2023). Huang et al. (2023) conducted a pivotal study in early-stage PD patients, demonstrating that gut dysbiosis emerges during the prodromal phase. Their findings elucidate two primary mechanistic pathways by which microbial imbalance impacts the nervous system. Firstly, the impairment of intestinal epithelial barrier function leads to increased intestinal permeability, allowing pro-inflammatory bacteria and their metabolic products, as well as inflammatory mediators, to directly act on the ENS (Procházková et al., 2024), promoting the abnormal aggregation of *α*-syn in the ENS and may spread to the CNS through the vagus nerve. Secondly, Gut microbiota dysbiosis disrupts BBB integrity (Papayannopoulos, 2018), directly inducing CNS inflammatory responses and pathological α-syn deposition, ultimately leading to dopaminergic neuronal death (Kuai et al., 2021). Notably, emerging evidence indicates that gut microbial dysbiosis reduces short-chain fatty acid (SCFA) production and alters neurotransmitter and hormonal homeostasis, particularly serotonin 5-HT signaling, consequently diminishing endogenous neuroprotective factor synthesis—a potential key contributor to dopaminergic neuron apoptosis in PD pathogenesis. In a cohort of 154 PD patients, reduced abundance of butyrate-producing bacteria and decreased levels of SCFAs were associated with increased mucosal permeability and systemic endotoxin exposure from gut microbiota (Keshavarzian et al., 2015). Recent investigations have consistently demonstrated reduced fecal SCFA concentrations in PD patients, with significant correlations observed between gut microbiota diversity, composition and levels of SCFAs, pro-inflammatory cytokines, and zonulin in stool samples (Aho et al., 2021).

#### 2.2.2 Factors contributing to gut microbiota dysbiosis

The composition of gut microbiota exhibits considerable interindividual variability, contingent upon intestinal physiological status (normobiosis vs. dysbiosis). Multiple extrinsic and intrinsic factors contribute to microbial ecosystem disruption, including but not limited to: Dietary patterns, Pressure, Aging, Birth method, and Lifestyle factors.

Ross et al. (2024) proposed that dietary patterns modulate PD progression through a microbiota-dependent mechanism: specific microbial metabolites either potentiate or suppress intestinal immune activation, consequently regulating neuroinflammatory processes in the CNS. Their findings support the clinical implementation of Mediterranean-style dietary regimens—characterized by high dietary fiber intake and polyphenol-rich foods as a potential disease-modifying strategy for PD management (Scheperjans et al., 2015). Clinical evidence demonstrates that dietary fiber intake through its bioactive metabolites exerts the role of regulating neuroinflammation in PD patients via microglial regulation, and selectively promoting the growth of beneficial commensals. These microbiota-derived SCFAs confer dual protective benefits through their established anti-inflammatory properties and ability to maintain BBB integrity (Makki et al., 2018; Cronin et al., 2021). A 2024 clinical study published in The Lancet Microbe (Kullberg et al., 2024) demonstrated a significant positive correlation between butyrate concentrations and intestinal barrier function in human subjects. Adherence to Mediterranean dietary patterns was associated with significantly increased fecal abundances of Bacteroides and Bifidobacterium genera, along with elevated SCFA concentrations—microbial signatures consistently identified as beneficial for PD patients (Garcia-Mantrana et al., 2018). A large-scale prospective cohort study of over 47,000 Swedish women (Ng A. S. L. et al., 2020) demonstrated that adherence to Mediterranean dietary patterns during midlife was significantly associated with reduced risk of PD development in later years. In a 12-month randomized, multicenter, single-blind, controlled trial involving 612 participants, Ghosh et al. (2020) demonstrated that enhanced adherence to the Mediterranean diet significantly modulates specific gut microbiota components associated with: Reduced risk of symptom progression; Improved cognitive performance and attenuated systemic inflammation. Their findings support the therapeutic potential of Mediterranean dietary patterns in chronic neurological disorders like PD, mediated through microbiota-derived mechanisms including enhanced SCFA production and potent anti-inflammatory effects, which may collectively mitigate disease risk and retard pathological progression. The study by García Mansilla et al. (2024) provides mechanistic evidence that SCFA ameliorate dysbiosis-associated gastrointestinal symptoms through three coordinated actions, including modulation of gut microbial ecology, enhancement of intestinal epithelial barrier integrity and suppression of pro-inflammatory immune responses. A recent investigation (Kawamoto et al., 2023) employing germ-free (GF) murine models has demonstrated that aged microbiota directly drive the accumulation of cellular senescence markers in ileal B lymphocytes, establishing a causal relationship between microbial aging and host the degeneration of immune system. Consequently, aging-induced intestinal inflammation and alterations in gut microbiota may impact the degeneration of the immune system and exacerbate the progression of neurodegenerative diseases.

**Figure 1:**
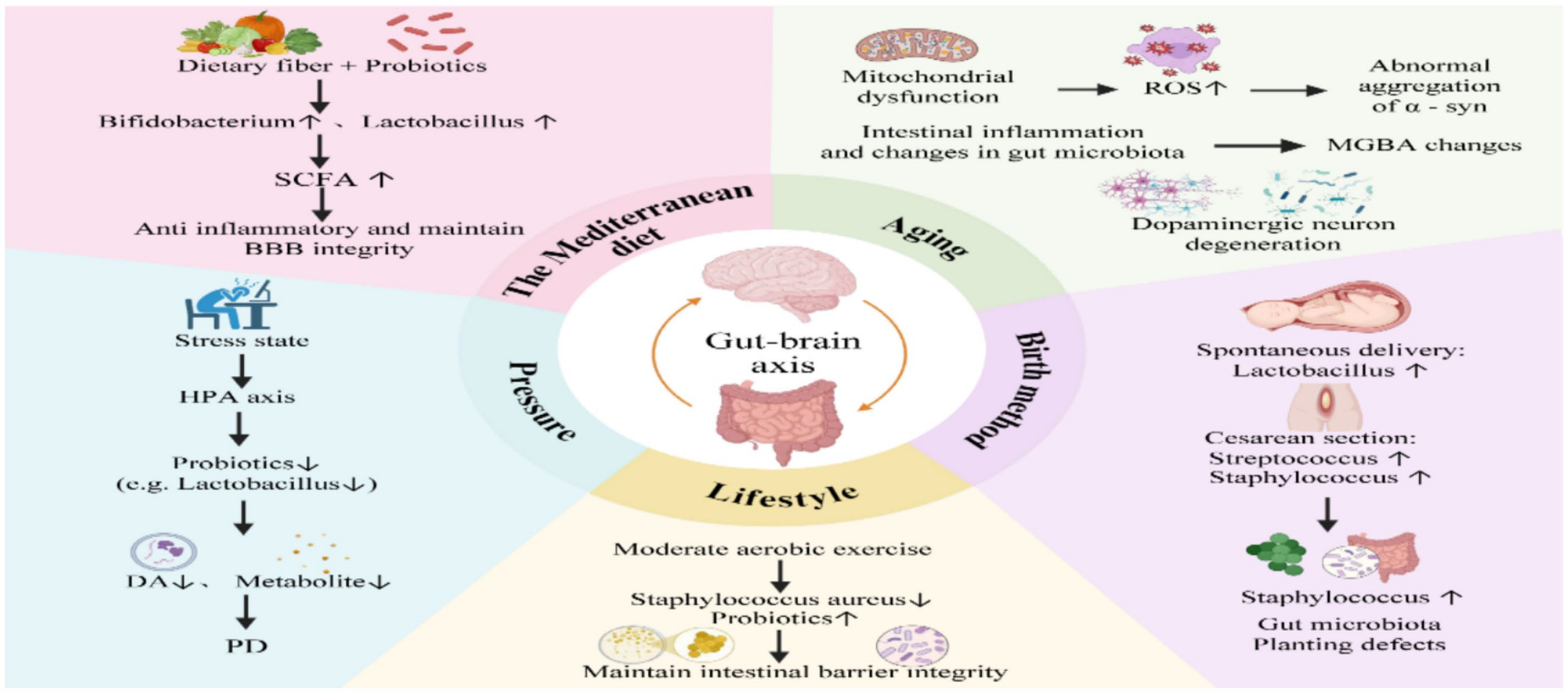
The mechanisms by which various factors affecting the gut microbiota contribute to the pathogenesis of Parkinson’s disease.

Foster et al. (2017) demonstrated in stressor-exposed animal models that psychological stress disrupts gut microbial composition, notably increasing *Lactobacillus plantarum* colonization. This microbial shift was associated with elevated striatal dopamine, serotonin, and their metabolites, ultimately contributing to PD. We hypothesize that excessive maternal stress during late pregnancy may elevate intra-abdominal pressure, potentially necessitating cesarean delivery due to compromised natural childbirth capacity. This iatrogenic intervention could subsequently increase Lactobacillus colonization and disrupt normal microbial succession in the neonatal gut, suggesting a potential bidirectional relationship between prenatal stress and delivery mode selection. Trancikova et al. (2012) demonstrated that vaginally delivered neonates acquire microbial communities resembling maternal vaginal microbiota, characterized by high Lactobacillus abundance. In contrast, cesarean-born infants exhibit microbiota profiles dominated by skin-associated bacteria, particularly Streptococcus and Staphylococcus species. Cesarean delivery may result in impaired microbial colonization of the neonatal gut, exerting long-term consequences on host metabolic programming and immune system development (O’Neill et al., 2020). Korpela et al. (2018) experimentally demonstrated that FMT not only restores the gut microbiome of cesarean-delivered infants, but also promotes microbial community development resembling that of vaginally born neonates. Importantly, this reconstituted microbiota maintains critical ecological functions, particularly in providing colonization resistance against pathogenic invaders. A murine model study (Xu et al., 2025) demonstrated that moderate exercise significantly reduces methicillin-resistant *Staphylococcus aureus* (MRSA) intestinal colonization while synergizing with probiotics to maintain intestinal barrier integrity and preserve gut homeostasis. Mahalakshmi et al. (2020) demonstrated through their investigation of exercise-induced neuroprotective mechanisms that moderate aerobic exercise ameliorates motor symptoms in PD patients via multiple pathways: Modulating neurotransmitter and energy metabolism, reducing oxidative stress, upregulating neurotrophic factors, and attenuating neuroinflammatory responses. Additionally, a healthy lifestyle decelerates aging processes and mitigates stress-related physiological burdens. Therefore, we hypothesize that there are intrinsic interconnections among the various factors influencing gut microbiota composition. Furthermore, several research groups have conducted in-depth investigations into extrinsic factors influencing gut microbiota. Their findings reveal a bidirectional relationship between intestinal flora and circadian rhythms that maintains host metabolic homeostasis. Disruption of circadian rhythms may increase intestinal permeability while altering both gut barrier function and microbial composition (Zou et al., 2022). Microbial metabolites play a pivotal bridging role in the MGBA. However, current studies investigating circadian rhythm effects on gut microbiota remain statistically underpowered due to insufficient sample sizes in subject selection (Figure 1).

#### 2.2.3 Alterations in gut microbiota composition are observed in patients with PD

Multiple PD studies have demonstrated that gut dysbiosis may induce intestinal inflammation and neuropathology through MGBA, ultimately accelerating disease progression (Table 1). The gut microbiota’s influence on the brain involves complex bidirectional communication, mediated primarily by bacterial metabolites that interact with both the host’s immune system and neurochemical pathways. Current clinical research on PD pathogenesis induced by gut dysbiosis primarily focuses on four mechanistic aspects: increased intestinal barrier permeability (Forsyth et al., 2011; Clairembault et al., 2015); neuroinflammation and oxidative stress in the brain (Pascual et al., 2021); molecular mimicry associated with *α*-syn pathology (Weiduschat et al., 2014; Caggiu et al., 2016; Miraglia and Colla, 2019); endocrine mechanisms triggered by various gut microbial metabolites (Yano et al., 2015). Species identified as pathogenic strains in PD pathology primarily include *Escherichia coli* (Chen et al., 2016; Sampson et al., 2020), lipopolysaccharide (Niu et al., 2020), Desulfovibrio (Huynh et al., 2023), *Lactobacillus plantarum* (Wang L. et al., 2022), and *Helicobacter pylori* (Pierantozzi et al., 2006). In 2024, Aburto and Cryan (2024) reported that gut microbiota and their metabolites regulate both the intestinal epithelial barrier and BBB through tight junction modulation, thereby preventing the translocation of harmful substances into the brain. The balance of microbial composition is referred to as a probiotic state. Bacteria have co-evolved with humans, establishing a symbiotic host-microbe relationship (Thursby and Juge, 2017). The gut microbiota plays essential roles in intestinal motility, barrier homeostasis, maintenance of intestinal integrity, modulation of the host immune system, and nutrient absorption and production. In PD, alterations in the gut microbial composition can lead to increased intestinal permeability. This allows the translocation of proinflammatory molecules and toxins into systemic circulation, ultimately reaching the brain where they exacerbate neuroinflammation and further compromise barrier function. This pathological cascade, commonly referred to as ‘leaky gut,’ establishes a vicious cycle of inflammation (Mulak and Bonaz, 2015). Emerging evidence suggests that PD-associated gut dysbiosis, particularly the depletion of SCFA-producing bacteria and concomitant enrichment of pathogenic species, correlates with increased intestinal permeability, immune activation, and pathological *α*-syn aggregation. Consequently, gut microbiota modulation represents a promising novel biomarker and therapeutic target for PD intervention (Cryan et al., 2019). Previous studies have consistently demonstrated alterations in gut microbiota composition in PD patients, characterized by reduced abundance of beneficial bacteria and concomitant elevation of pro-inflammatory bacterial taxa (e.g., Enterobacteriaceae). These microbial alterations are postulated to contribute to PD pathogenesis through multiple mechanisms, including neuroinflammation and aberrant protein aggregation (Keshavarzian et al., 2015). A clinical trial utilizing metagenomic sequencing data from the RevMan 5.2 database conducted a meta-analysis of 498 PD patients across 10 studies. The analysis revealed that elevated *Helicobacter pylori* abundance correlates with symptom exacerbation in PD patients. Notably, *H. pylori* eradication therapy was found to prolong L-dopa onset time, improve stride length in bradykinesia indices, and significantly enhance UPDRS-III scores (Bai and Li, 2021). Jin et al. (2019) demonstrated a significant positive correlation between Prevotella abundance and PD severity, while observing an inverse relationship with Zurichbacter levels. Furthermore, their findings indicate that Prevotella may promote pathological α-syn aggregation, compromise intestinal barrier integrity, and disrupt gut homeostasis, thereby potentially exacerbating PD progression. Current research has established that Prevotella, while being a common commensal bacterium in the human gastrointestinal tract, demonstrates reduced abundance in PD patients—particularly in those with more rapid disease progression. This finding underscores the potential significance of this bacterial genus in PD pathogenesis. To accurately capture the dynamic changes in microbial communities throughout disease progression, extended follow-up periods may be required. A microbiome analysis of 64 PD patients revealed a significant reduction in the abundance of beneficial bacteria such as Lachnospiraceae compared to healthy controls. This pattern is associated with intestinal inflammation and may contribute to the development of neuropsychiatric symptoms, particularly depression (Aho et al., 2019). A multinational study conducted 16S rRNA sequencing (V1–V3 regions) of fecal microbiota from 72 PD patients and 72 healthy controls. The analysis revealed a significant reduction in Prevotella abundance alongside increased Enterobacteriaceae levels in PD patients. Notably, the elevated Enterobacteriaceae abundance showed a positive correlation with the severity of postural instability and gait difficulty (PIGD) in PD subjects (Scheperjans et al., 2015). Notably, this study found that the reduced abundance of Prevotella could not account for the constipation symptoms observed in PD patients. A cohort study involving 197 PD patients and 103 healthy controls revealed significant alterations in gut microbiota composition. Specifically, PD patients exhibited increased abundances of Christensenella and Akkermansia, along with decreased levels of Lachnospira and Faecalibacterium. The researchers hypothesized that these microbial changes may correlate with stool consistency and constipation severity in PD (Cirstea et al., 2020). However, dietary variations and interference from PD medication regimens remain unavoidable confounding factors in these clinical investigations. Furthermore, Barichella et al. (2019) conducted an in-depth investigation comparing PD with atypical parkinsonian syndromes. Their findings revealed that reduced Lachnospiraceae abundance was the sole distinguishing feature between *de novo* PD patients and healthy controls, while Lactobacillus and Christensenella exhibited increased abundance compared to controls. Notably, Multiple System Atrophy (MSA) and Progressive Supranuclear Palsy (PSP) patients shared similar microbial alterations with PD, with several exceptions: MSA patients showed no reduction in Lachnospiraceae but displayed decreased Prevotellaceae; PSP patients maintained normal Lactobacillaceae levels but demonstrated reduced Streptococcaceae. These specific bacterial taxonomic changes were significantly associated with cognitive impairment and gastrointestinal dysfunction in PD patients. These findings suggest that modulating gut microbiota composition may alleviate PD-related symptoms, providing compelling evidence for potential therapeutic strategies. However, the correlation between microbial relative abundance and disease progression in PD patients remains inconsistent, likely due to confounding factors including methodological variations, interindividual differences, ethnic disparities, and medication effects. For instance, geographically distinct dietary patterns, a known determinant of gut microbial composition—may account for significant cross-regional variations in microbiome profiles (Aho et al., 2019).

**Table 1:** Alterations in the gut microbiota of Parkinson’s disease patients and their clinical correlations.

Study participants	Alterations in gut microbiota	Mechanisms	Clinical relevance	Clinical limitations	References
498 Patients with Parkinson’s disease	*Helicobacter pylori*↑	Interference with levodopa absorption	Motor symptoms UPDRS-III scores Gastrointestinal symptoms	Medical ethics restrictions No statistically significant improvement in muscle rigidity Insufficient number of randomized controlled trials (RCTs) Subject to individual variability limitations	Bai and Li (2021)
72 Patients with Parkinson’s disease	Prevotella↓ Zurichibacter↓	Promotion of α-synuclein (α-syn) pathological aggregation; Disruption of intestinal barrier integrity and gut homeostasis	Parkinson’s disease severity	Pharmacological interference in Parkinson’s disease Ethnic origin, geography, age, genetics, and spousal status	Jin et al. (2019)
64 Patients with Parkinson’s disease	Lachnospira↓ Prevotella↓ Nodococcus↓ Bifidobacterium↓	Underlying mechanism remains unclear	Parkinson’s disease severity Psychiatric symptoms (e.g., depression) Intestinal inflammation	Diet, family history, statin use, and total SCS-PD score Confounding factors including NMS quest Limited sample size	Aho et al. (2019)
72 Patients with Parkinson’s disease	Prevotella↓ Enterobacter↓	Modulation of enteric neuron activity; Modulation of α-synuclein secretion	Severity of postural instability and gait difficulty (PIGD)	Participants collected samples at home Gender ratio, body mass index (BMI), comorbidities, medications, and deep brain stimulation (DBS) therapy	Scheperjans et al. (2015)
197 Patients with Parkinson’s disease	Christensenella↑ Akkermansia↑ Lachnospira↓ Prevotella↓ Nodococcus↓ Bifidobacterium↓ Faecalibacterium↓	Two byproducts of protein degradation (p-Cresol and phenylacetylglutamine); Increased fecal consistency and firmness	Fecal consistency Severity of constipation Gastrointestinal function (e.g., irritable bowel syndrome, IBS)	Diet and Parkinson’s disease medication interactions Cross-sectional study Sex ratio	Cirstea et al. (2020)
193 Patients with Parkinson’s disease 22 Patients with progressive supranuclear palsy 22 Patients with multiple system atrophy (MSA)	Lachnospira↓ Christensenella↑ Akkermansia↑ Lachnospira↓ Prevotella↓ Nodococcus↓ Bifidobacterium↓ Faecalibacterium↓ Lactobacillus↑	Production of short-chain fatty acids ↓; Impairment of intestinal epithelial barrier integrity	Cognitive impairment gait dysfunction Postural instability	Cross-sectional study Sex ratio	Barichella et al. (2019)

A growing body of evidence suggests that gut microbiota analysis holds promise as a valuable tool for early detection of PD. Although gut microbiota cannot yet serve as an independent clinical diagnostic criterion, it shows significant potential in complementing existing diagnostic methods, identifying high-risk individuals, and enabling ultra-early interventions. Gastrointestinal symptoms typically emerge approximately 10–20 years before motor manifestations of PD, providing a critical therapeutic window. According to the MGBA theory, alterations in gut microbiota may capture early pathological signals during initial brain lesions, suggesting microbiota-targeted interventions could effectively prevent PD progression. The gut microbiota is directly implicated in early PD pathogenesis. Certain gut microbes can produce specific amyloid proteins that cross-seed misfolding of human *α*-syn. Dysbiosis in PD patients compromises intestinal barrier integrity—a condition termed “leaky gut,” which facilitating the translocation of endotoxins and inflammatory factors that trigger neuroinflammation. Moreover, gut microbiota generates metabolites such as SCFA and serotonin. Butyrate, a key SCFA, exerts anti-inflammatory effects, maintains barrier function, and provides neuroprotection. Early PD patients typically exhibit reduced butyrate-producing bacteria and increased pro-inflammatory microbes like Enterobacteriaceae, contributing to the development of characteristic motor symptoms. Therefore, targeted modulation of the gut microbiota represents a promising strategy for preventing and treating early-stage PD. A recent study (Li et al., 2025) has utilized machine learning models to explore gut microbiota as a novel biomarker for PD. By analyzing specific microbial profiles, these models construct a “microbial risk score” to assist in PD diagnosis and early-risk stratification. For populations with a family history of PD or carrying PD-risk genes such as LRRK2, monitoring gut microbiota may help identify individuals who are beginning to develop pathological changes. Importantly, if PD risk can be detected via microbiota analysis prior to the onset of motor symptoms, early and effective interventions could be implemented to prevent or delay disease progression. A study led by Professor Jintai Yu (Wu et al., 2025), an elderly patient diagnosed with early-stage PD in October 2020 received a gut-immune-microecology intervention. Following treatment, the patient exhibited improved walking capacity with a lighter gait, reduced tremors, enhanced fine motor skills, better mental state and appetite, and complete resolution of constipation. Additionally, gqut microbiota diversity increased, and neurotransmitter levels returned to normal.

#### 2.2.4 The impact of gut microbiota on inducing PD models

PD bio-models are widely employed in preclinical research to investigate disease etiology and therapeutic efficacy by replicating specific PD features in animals or cell cultures. Common models include the MPTP-induced, 6-OHDA-lesioned, rotenone-based, and genetic models. Both genetic PD models and MPTP-induced paradigms serve as excellent platforms for studying gut microbiota’s influence on PD pathogenesis. This section will therefore focus on elucidating the mechanisms through which gut microbiota modulates these two model systems. Substantial evidence confirms that gut microbiota plays a critical dual role as both a “modulator” and “amplifier” in these experimental paradigms.

##### 2.2.4.1 Impact of gut microbiota on genetic models of PD model

It commonly referred to as the “*α*-syn overexpression model,” this approach utilizes viral vectors or transgenic technologies to induce overexpression of human mutant or wild-type α-synuclein in rodents. In 2024, Sandra et al. (Munoz-Pinto et al., 2024) demonstrated that transplanting gut microbiota from Parkinson’s disease patients into wild-type male mice induced intestinal inflammation and α-synuclein aggregation within weeks, subsequently progressing to systemic inflammation, impaired intestinal barrier function, dopaminergic neuron loss in the substantia nigra, mitochondrial dysfunction, and PD-specific motor deficits. Similarly, Weidong Le et al. (Yang H. et al., 2024) reported that FMT from PD patients to A53T transgenic mice not only altered gut microbiota composition but also modified fecal metabolic profiles, as revealed by 16S rRNA gene sequencing and metabolomic analyses. We summarize the mechanisms by which gut microbiota influences genetic PD models into two key aspects. First, gut microbiota remodeling is associated with reduced Th17 cell homeostasis, directly contributing to intestinal inflammation; Second, PD transplant mice exhibit decreased CD4 + T cells and increased pro-inflammatory cells in peripheral blood, which subsequently migrate via the vagal neurons to activate microglia in the brain, thereby accelerating α-syn aggregation, mitochondrial dysfunction, and dopaminergic neuron degeneration.

##### 2.2.4.2 Impact of gut microbiota on MPTP-induced PD model

The MPTP-induced model, as a classical neurotoxin paradigm, rapidly crosses the blood–brain barrier following systemic administration. It is metabolized in astrocytes to MPDP+, which subsequently converts to the active toxin MPP+. This metabolite is selectively taken up by dopaminergic neurons via dopamine transporters (DAT) and accumulates intracellularly. MPP + is then stored in mitochondria in an ATP-dependent manner, where it inhibits complex I of the electron transport chain, leading to energy failure and neuronal degeneration (Mustapha and Mat Taib, 2021). Research (Qiao et al., 2023) has demonstrated that FMT pretreatment in MPTP-induced PD models can enhance neurogenesis in aged mice, improve motor function, and restore dopaminergic neurons and neurotransmitters. FMT supplementation with aged microbiota ameliorates motor deficits, mitigates the loss of dopaminergic neurons, attenuates striatal dopamine depletion, and elevates striatal serotonin levels in MPTP-induced Parkinson’s disease model mice. Furthermore, when administered prior to MPTP intoxication, FMT derived from aged donors restores gut microbial homeostasis, enhances hippocampal neurogenesis, and reduces fecal SCFA concentrations in these mice.

Current clinical practice lacks standardized guidelines for managing PD-related gastrointestinal dysfunction. We propose that microbiota-targeted therapeutic approaches primarily encompass two strategies: selective microbial modulation and FMT. The former demonstrates better safety profiles albeit with limited efficacy, while the latter enables precise microbial reconstitution with superior therapeutic potential, albeit accompanied by higher clinical risks and implementation challenges. Given these considerations, developing innovative therapeutic targets to effectively alleviate both motor and non-motor symptoms of PD holds significant clinical value for slowing disease progression and improving patients’ quality of life.

### 2.3 The MGBA operates through multiple integrated pathways

**Figure 2:**
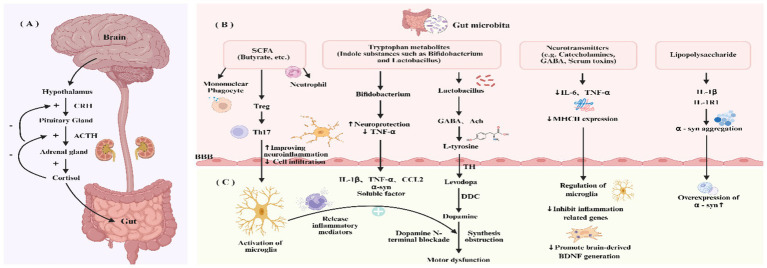
Mechanisms of microbiota gut brain axis communication. **(A)** The gut microbiota interacts intensively with the nervous system, segmentally regulating intestinal motility, secretion, and signal transduction. Under stress conditions, this regulatory network operates through the hypothalamic–pituitary–adrenal axis via a three-tiered mechanism. **(B)** Gut microbiota-derived metabolites comprise both beneficial metabolites (short-chain fatty acids, tryptophan metabolites, neurotransmitters) and harmful metabolites (lipopolysaccharides), which collectively regulate intestinal substance metabolism through immune and molecular mechanisms. **(C)** Gut microbiota-derived metabolites traverse physiological barriers and enter the systemic circulation, ultimately crossing the blood–brain barrier. This process activates microglia, triggering the release of inflammatory mediators that induce dopaminergic neurodegeneration or cell death, culminating in the characteristic clinical manifestations of Parkinson’s disease.

The bidirectional communication between the human gut microbiota and the CNS, termed the MGBA, involves complex interactions across multiple pathways. The MGBA modulates host brain activity, behavior, and neurodevelopment through neural networks (e.g., enteric and central neural circuits), the neuroendocrine HPA axis, and immune signaling (Figure 2). This crosstalk is facilitated by vagal, sympathetic, and enteric neural fibers, with regulatory input from the HPA axis.

#### 2.3.1 Neural pathways of the microbiota-gut-brain axis (MGBA)

The gut microbiota interacts bidirectionally with the nervous system to maintain intestinal homeostasis. The gastrointestinal tract is innervated by extrinsic sympathetic and vagal nerves, as well as the intrinsic ENS. Under physiological conditions, gut motility and secretory functions are primarily regulated by the ENS. During homeostatic imbalance, however, these processes become predominantly modulated by central autonomic pathways (Sampson et al., 2016). The vagus nerve serves as the primary direct neural pathway connecting the gut and brain, regulating intestinal motility, secretion, and sensory signal transmission. Through vagal and spinal efferent projections to the intestinal mucosa, this system directly and indirectly coordinates with the ENS to maintain gastrointestinal homeostasis. This integrated neural network modulates enteric neuronal activity, ultimately influencing gastrointestinal physiology, local immune function, and gut microbiota composition (Furness et al., 2014). Previous studies have demonstrated that specific gut microbiota modulate brain function through vagal nerve fibers. For instance, in mice, oral treatment with *Lactobacillus rhamnosus* reduced anxiety- and depression-related behaviors via the vagal neurons, whereas *Campylobacter jejuni* infection conversely promoted anxious behaviors. These effects are mediated through microbial stimulation of enteroendocrine cells to release glutamate as a neurotransmitter (Goehler et al., 2005). Kaelberer et al. (2018) recently identified a specialized subset of enteroendocrine cells (EECs), termed neuropods, that form glutamatergic synapses with vagal afferents, establishing a direct luminal-brainstem connection. Vagotomy studies in rodent models demonstrate that ablation of this pathway induces functional alterations in neural circuits and behaviors associated with neuropsychiatric disorders. Recent studies have explored vagal neurons stimulation (VNS) as a therapeutic intervention for neurodegenerative disorders. The underlying mechanism primarily involves enhanced modulation of specific neurotransmitters, such as acetylcholine, *γ*-aminobutyric acid (Yunes et al., 2016), dopamine, norepinephrine, and serotonin and other neuroactive substances, such as brain-derived neurotrophic factor (Zhou et al., 2019) and cytokines (Lespérance et al., 2024). The ENS, an integral component of the peripheral nervous system (PNS), occupies a critical interface between the host and gut microbiota. This complex neural network, often referred to as the ‘second brain,’ autonomously regulates gastrointestinal functions while maintaining bidirectional communication with the CNS (Schneider et al., 2019). The ENS comprises two principal ganglionated plexuses: the myenteric plexus (Auerbach’s plexus) situated between the longitudinal and circular muscle layers, and the submucosal plexus (Meissner’s plexus) located between the circular muscle layer and mucosa. The ENS orchestrates gastrointestinal motor and secretory functions, coordinating peristalsis while regulating the synthesis and release of digestive enzymes and gut hormones (Furness, 2012). Emerging evidence indicates that the development and functionality of the ENS are modulated by both the gut microbiota and host immune system. Beyond its crucial role in maintaining gastrointestinal homeostasis, the ENS serves as a pivotal relay station within the MGBA, integrating bidirectional communication between microbial signals and central neural processes (Moțățăianu et al., 2023). Recent studies demonstrate that alterations in the ENS may serve as key drivers of gut microbial dysbiosis through modulation of intestinal transit, barrier permeability, and luminal PH. Conversely, the gut microbiota also exerts significant influence on ENS growth and development, establishing a bidirectional regulatory relationship (Baumler et al., 2022). During critical developmental windows, dynamic host-microbiota interactions may elevate susceptibility to neurodevelopmental disorders and exert long-term neuronal consequences. These findings suggest that ENS dysfunction could contribute to the pathogenesis of various neurodegenerative diseases. Consequently, elucidating the mechanistic interplay between the ENS and host microbiota will facilitate the development of targeted therapeutic strategies.

#### 2.3.2 Immunological pathways of the MGBA

PD patients exhibit gut microbiota alterations with pro-inflammatory potential, as intestinal microbial communities serve as crucial regulators of local immune responses. Keshavarzian et al. documented significant compositional changes in both mucosa-associated and fecal microbiota in PD patients compared to healthy controls. The observed dysbiosis may propagate inflammation through multiple mechanisms: compromising intestinal barrier integrity, triggering immune activation, and inducing systemic inflammatory responses—collectively exacerbating PD pathogenesis (Ivanov and Honda, 2012; Nishio and Honda, 2012; Caricilli, 2014). Conversely, the brain can modulate inflammatory signaling along the MGBA through efferent neural pathways, establishing a self-perpetuating feedback loop. This bidirectional communication enables the CNS to dynamically respond to peripheral immune challenges while simultaneously regulating gut inflammatory tone (Aho et al., 2021). The immune system operates through two principal arms: innate immunity and adaptive immunity, which we will delineate sequentially.

##### 2.3.2.1 Innate immunity

Molecules involved in innate immunity, such as toll-like receptors (TLRs), cytokines, complement families, and adaptive immunity-related molecules like antibody receptors and major histocompatibility complex (MHC), are produced in the brain and play key regulatory roles in CNS development. There exists a complex network connection among the immune system, CNS, and intestinal microbiota. Specifically, the interaction between intestinal microbiota and the immune system regulates local immunity in the intestine through microorganisms themselves, microbial derivatives, such as SCFA, secondary bile acids, and amino acid metabolites and other bioactive molecule, such as microbe-associated molecular patterns (MAMPs), influences the CNS through systemic circulation, and exerts regulatory effects mediated by microglia (Liew and Kubes, 2019). Microglia, the resident innate immune cells of the CNS, play a crucial neuroprotective role through debris clearance and homeostatic maintenance. Compelling evidence demonstrates that *α*-syn aggregates can activate microglia via both Toll-like receptor 4 (TLR4) and Toll-like receptor 2 (TLR2) pathways, triggering subsequent neurotoxic effects on dopaminergic and other vulnerable neuronal populations (Duffy et al., 2018). Accumulating evidence demonstrates that gut-derived inflammation, characterized by intestinal inflammatory responses and dysbiosis-associated immune activation, contributes to the pathogenesis of PD (Tan et al., 2020). The gut microbiota modulates immune responses through chemokine and cytokine regulation, primarily via the following mechanisms: First, Proinflammatory cytokines enter systemic circulation and traverse the compromised BBB; Second, these mediators recruit peripheral immune cells that infiltrate the CNS; Third, the infiltrating immune cells interact with resident microglia and astrocytes, establishing a self-perpetuating cycle of neuroinflammation. Pathway-based analyses reveal significant associations between PD risk and genes involved in leukocyte/lymphocyte activity regulation and cytokine-mediated signaling pathways (Southwell et al., 2016). Gut microbiota and their metabolites can trigger *α*-syn-dependent microglial activation, subsequently promoting the release of proinflammatory cytokines that induce dopaminergic neuronal degeneration, ultimately contributing to motor dysfunction in PD patients. Studies using α-syn-overexpressing murine models demonstrate that microbial modulation influences both α-syn aggregation and the impairment of insoluble protein aggregate clearance pathways (Sampson et al., 2016). We emphasize the pivotal role of microglia in PD models. Beyond their previously discussed mechanisms in innate immunity, microglia indirectly engage with the peripheral immune system, thereby forming a crucial bridge between innate and adaptive immunity. First, activated microglia release signaling molecules—including cytokines/chemokines (e.g., IL-1β, TNF-α, CCL2), neurodegenerative signals such as α-syn, and soluble factors—that directly recruit and activate peripheral immune cells. This cascade triggers antigen-specific immune responses against cerebral antigens, amplifying neurotoxic signaling and neuronal injury. Subsequently, peripheral antigen-presenting cells capture these signals, activating T and B cells to produce pro-inflammatory cytokines that drive systemic neuroinflammation (Sampson et al., 2016). Notably, depleting gut microbiota via antibiotic treatment in MPTP-induced mice not only reduces dopaminergic neuron loss but also significantly suppresses microglial activation in the substantia nigra. Furthermore, gut microbiota can polarize macrophages into pro-inflammatory M1 phenotype or anti-inflammatory M2 phenotype, stimulating the secretion of corresponding cytokines (e.g., TNF-α/IL-1β/IL-6 for M1; IL-4/IL-13 for M2). Notably, these peripherally activated macrophages may migrate to the brain and differentiate into microglia, ultimately exhibiting either neuroprotective or neurotoxic functions. This macrophage-microglia axis plays a pivotal role in maintaining intestinal barrier integrity and gut homeostasis.

##### 2.3.2.2 Adaptive immunity

**Figure 3:**
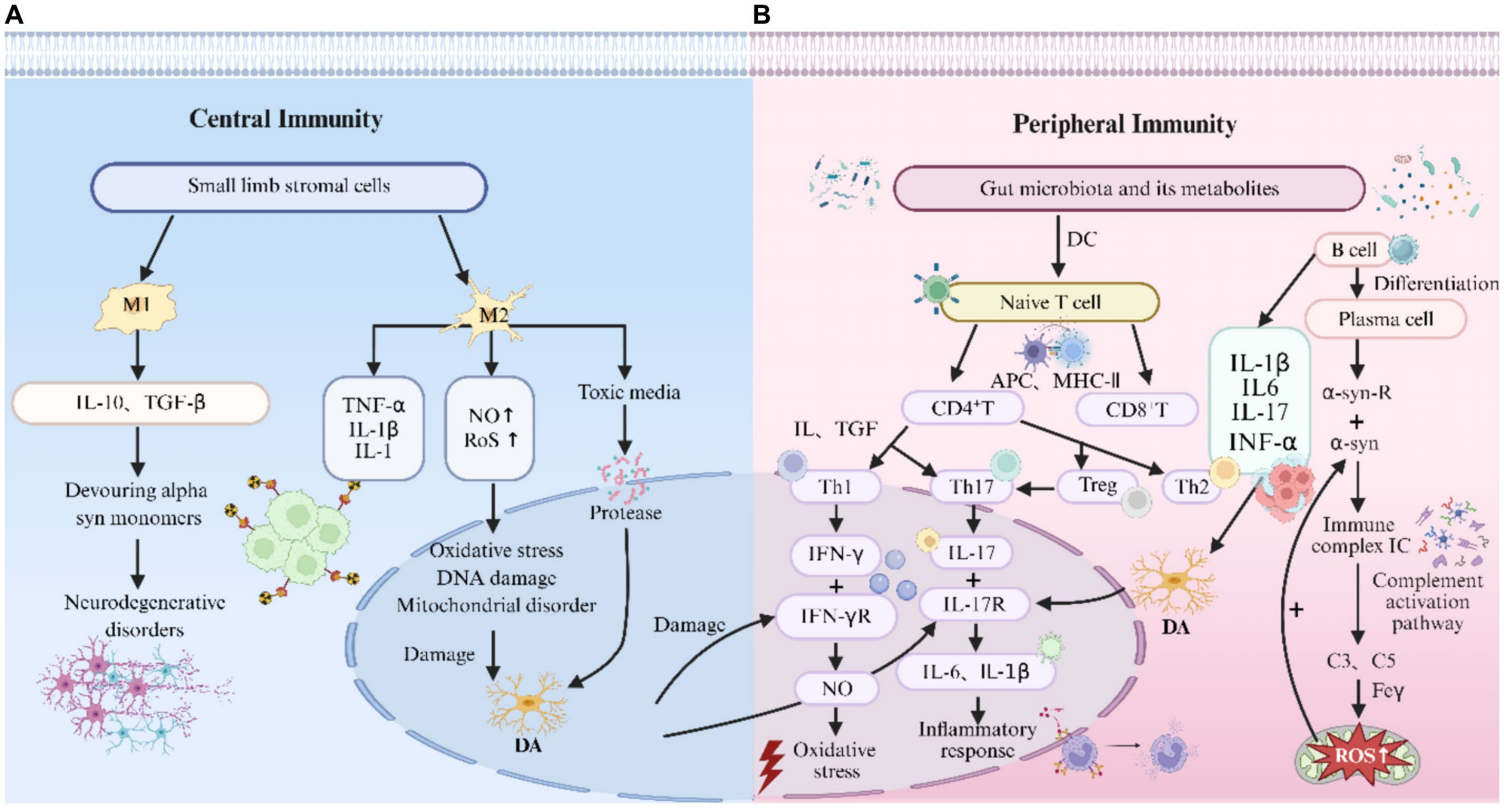
Immune-inflammatory pathways in Parkinson’s disease. **(A)** Central immune pathways in Parkinson’s disease: Microglia: As sentinel immune cells of the central nervous system, microglia drive dopaminergic neurodegeneration through the neurotoxic mechanisms of M1 phenotype and the inflammation-amplifying mechanisms of M2 phenotype. M1 microglia release abundant pro-inflammatory factors, including TNF-α (which binds TNF-R1 to activate apoptotic pathways) and IL-1β (which suppresses BDNF expression). They also generate substantial NO and reactive ROS, inducing oxidative stress, DNA damage, and mitochondrial dysfunction to directly impair dopaminergic neurons. Additionally, they secrete cytotoxic mediators such as proteases, further damaging neuronal integrity. M2 microglia, while traditionally considered neuroprotective, may paradoxically contribute to pathology by secreting pro-inflammatory factors like IL-10 and TGF-β, and by phagocytosing α-syn monomers in a manner that potentially facilitates neurodegenerative processes. **(B)** Peripheral immune pathways in Parkinson’s disease: T cells: Gut microbiota and their metabolites are recognized by dendritic cells, which subsequently activate naïve T cells via MHC II-mediated antigen presentation to CD4+ T cells, driving their differentiation into pro-inflammatory Th1/Th17 subsets and anti-inflammatory Th2/Treg subsets. Th1 cells secrete IFN-*γ*, which binds to IFN-γ receptors on dopaminergic neurons, inducing substantial nitric oxide (NO) production and triggering oxidative stress-mediated damage. Th17 cells release IL-17 that engages IL-17 receptors on dopaminergic neurons, increasing blood–brain barrier permeability and promoting the release of IL-6 and IL-1β, thereby exacerbating the cerebral inflammatory microenvironment. B cells: Upon differentiation into plasma cells, B cells secrete substantial quantities of α-synuclein-specific autoantibodies. These autoantibodies bind to α-synuclein deposits on dopaminergic neuronal surfaces, forming immune complexes that activate the classical complement pathway. This cascade triggers cleavage of C3 and C5, generating complement fragments that not only directly disrupt neuronal membrane integrity but also induce intracellular ROS accumulation via neuronal Fcγ receptors, thereby accelerating abnormal α-synuclein aggregation.

The gut microbiota and their metabolites serve as diverse exogenous antigens recognizable by dendritic cells (DCs), which subsequently prime naïve T cell activation and differentiation into CD4+ and CD8+ T cell subsets. Brochard et al. (2008) demonstrated that specific gut bacterial components may induce the formation of intestinal α-syn inclusions, which potentially act as autoantigen sources driving CD4+ T cell responses in PD. Furthermore, gut microbiota directly modulate CD4+ T cell polarization, promoting both pro-inflammatory (Th1/Th17) and anti-inflammatory (Treg/Th2) phenotypes. Regulatory T cells (Tregs) exhibit potent immunosuppressive capabilities that critically prevent autoimmune reactions and excessive inflammatory responses. Previous studies have established that SCFA produced by Clostridium species participate in the activation and proliferation of regulatory T cells (Tregs). These microbial metabolites enhance intestinal barrier integrity, thereby preventing pathogen translocation and mitigating systemic inflammation. Lee and Kim (2017) elucidated the imbalance between T cell-mediated immunity and proinflammatory responses in PD by analyzing the induction mechanisms of Th1/Th17 cells and suppression of Th2/Treg cells. These polarized T cell subsets secrete distinct proinflammatory cytokines that enter systemic circulation, cross the BBB, and interact with microglia to drive neuroinflammation. Consequently, broad-spectrum T cell inhibitors may confer neuroprotection by dual mechanisms: Directly suppressing pathogenic T cell proliferation and indirectly promoting immune tolerance through regulatory T cell induction. A 2023 Nature study (Park et al., 2023) provided compelling evidence that co-transplantation of autologous regulatory T (Treg) cells effectively ameliorates neuroinflammation in PD by suppressing peripheral immune cell infiltration and reducing graft mRNA degradation, offering a novel therapeutic strategy. Notably, environmental alterations or genetic predisposition may induce microbial dysbiosis, leading to increased Th1/Th17 cells and decreased Treg cells/IgA production, thereby predisposing to neurodegenerative disorders including PD. A recent Nature study (White et al., 2025) further elucidated the mechanisms by which CD4 + T cells mediate neuroinflammation via the MGBA. The authors demonstrated that peripherally activated CD4 + T cells infiltrate the CNS where they undergo reactivation through molecular mimicry of host antigens. These T cells subsequently activate microglia through both IL-23 receptor-dependent and GM-CSF-dependent pathways, ultimately driving neuroinflammatory cascades in PD. Furthermore, gut microbiota can stimulate B cells to produce diverse immunoglobulins (IgA, IgM, IgG, IgD, and IgE). These B cell-derived immunoglobulins may infiltrate the central nervous system and contribute to PD progression through neuroimmune interactions (Li et al., 2020). Wesemann et al. demonstrated that germ-free mice co-housed with conventionally raised counterparts exhibited significantly elevated pro-B cell populations in the small intestinal lamina propria at 3–4 weeks of age. This study establishes that gut microbial variability exerts deterministic effects on B cell development. Consequently, immunoglobulin-stimulating gut bacteria play an indispensable role in shaping the repertoire of B cell antigen receptors during colonization (Figure 3 provides a detailed depiction of the immune-inflammatory pathways involved in Parkinson’s disease).

Consequently, immune-targeted therapies for PD are progressively being translated into clinical applications, including both *α*-syn-directed immunotherapy and interventions targeting immune mediators (Fleming et al., 2022).

#### 2.3.3 Endocrine pathways of the MGBA

The gut microbiota and the HPA axis engage in bidirectional communication through neuroendocrine signaling pathways that regulate stress responses. During stress, hypothalamic secretion of corticotropin-releasing hormone (CRH) stimulates anterior pituitary release of adrenocorticotropic hormone (ACTH), which subsequently induces adrenal cortisol production. Elevated cortisol levels modulate gut microbial composition by altering intestinal microenvironment and immune responses, thereby maintaining intestinal homeostasis. Conversely, gut dysbiosis can reciprocally disrupt HPA axis function. Accumulating evidence indicates that dysfunction of the MGBA may contribute to irritable bowel syndrome, neurobehavioral abnormalities, and neurodegenerative disorders. Furthermore, gut microbiota-derived metabolites, neurotransmitters, and neuromodulators collectively participate in systemic metabolic regulation (Cussotto et al., 2018). The key microbial-derived metabolites include butyrate (GABA), serotonin, dopamine, norepinephrine, acetylcholine, histamine, secondary bile acids, 4-ethylphenyl sulfate (4EPS), and SCFA. Through interactions with the enteroendocrine system—comprised of diverse EECs producing glucagon-like peptide-1 (GLP-1), peptide YY (PYY), cholecystokinin (CCK), substance P, and 5-hydroxytryptamine (5-HT)—the gut microbiota critically regulate neurodevelopmental processes (Gribble and Reimann, 2016). Specific gut microbiota enhance tryptophan hydroxylase 1 (TPH1) expression through microbial metabolites such as secondary bile acids and SCFAs, thereby promoting 5-HT biosynthesis in ECCs (Reigstad et al., 2014). Emerging evidence, though not yet conclusive, suggests that lipopolysaccharide (LPS) may exert its effects through Toll-like receptor 4 (TLR4)-mediated pathways (Wei et al., 2024). Notably, peripheral 5-HT cannot cross the BBB. However, recent studies (Bonaz et al., 2018) have identified the presence of 5-HT3 and 5-HT4 receptors on vagal afferents, suggesting a potential indirect mechanism through which 5-HT may modulate CNS activity via vagal neurons signaling. While metabolites like 5-HT cannot directly access the vagal neurons or cross the BBB, microbial-derived metabolites interact with both vagal afferents and the ENS to modulate gut sensation. Notably, recent discoveries (Magistrelli et al., 2019) have identified direct neuroepithelial connections between EECs and vagal neurons through specialized neuropod cells. These cells form synaptic contacts with vagal terminals, establishing a novel rapid gut-to-brain signaling pathway.

## 3 Application of FMT and oral probiotic products in the treatment and prevention of PD

As evidenced above, gut microbiota modulation represents a novel therapeutic strategy for PD prevention and treatment. Dietary interventions, probiotic supplementation, and FMT currently rank as the three most promising approaches. These modalities exert beneficial effects by enriching commensal bacteria, suppressing pathogenic species, and modulating microbial metabolite production. Notably, SCFA may enhance BBB integrity while significantly mitigating neuroinflammation and PD risk. Current research confirms that probiotics and their constituents can effectively prevent and ameliorate PD symptoms via the MGBA, primarily through Gut microbiome stabilization, Anti-inflammatory and Antioxidant effects; Upregulation of neurotrophic factors (Magistrelli et al., 2019). A 2019 randomized, double-blind, placebo-controlled clinical trial (Tamtaji et al., 2019) involving 60 PD patients demonstrated that 12-week supplementation with a probiotic consortium, containing (*Lactobacillus acidophilus*, *Bifidobacterium bifidum*, *Lactobacillus reuteri*, and *Lactobacillus fermentum*) significantly improved motor function, insulin metabolism, and oxidative stress parameters. However, substantial heterogeneity in probiotic study designs and insufficient correlation with baseline host microbiota composition have limited definitive conclusions. Additional challenges include slow onset of action, narrow therapeutic windows, and poor controllability of probiotic interventions in humans. Several clinical trials have demonstrated that probiotic supplementation can alleviate constipation, improve bowel habits, and enhance MDS-UPDRS scores in PD patients, thereby ameliorating both motor and non-motor symptoms. The diversity of methodologies in probiotic research, coupled with insufficient correlation with the host’s native microbiota composition, complicates the derivation of definitive conclusions. Furthermore, probiotic interventions in humans exhibit slow efficacy, limited scope of action, and low controllability. Although probiotics offer potential benefits, inadequate administration may lead to unintended consequences—including detrimental metabolic activities, insufficient immune responses, and enhanced expression of antibiotic resistance genes—potentially resulting in systemic infections and compromised intestinal barrier integrity (Doron and Snydman, 2015). Consequently, recent research has increasingly focused on FMT as a more targeted approach for PD microbiome modulation.

### 3.1 FMT

**Figure 4:**
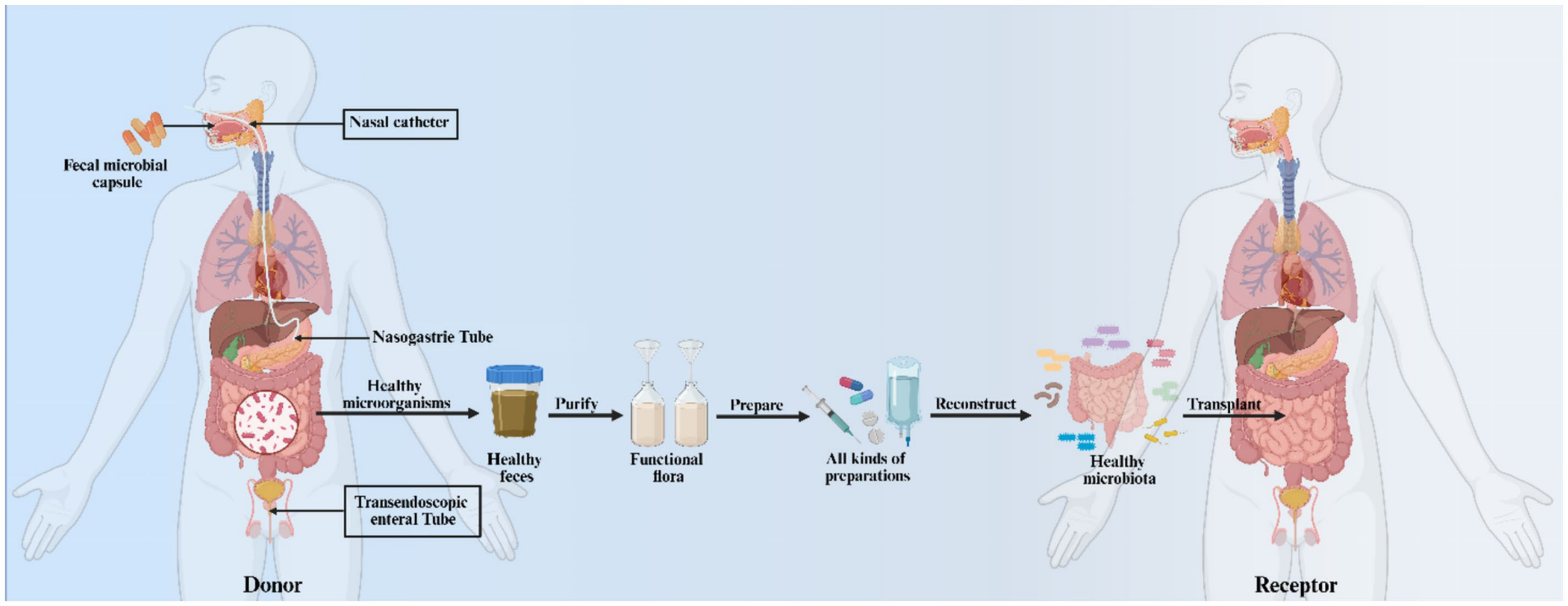
Overview of fecal microbiota transplantation: Methodology and mechanisms: Fecal microbiota transplantation (FMT), as the most effective method for reconstructing gut microbiota ecosystems, involves transferring functional microbial communities from healthy donors’ feces into the gastrointestinal tract of PD patients to establish new gut flora and achieve therapeutic effects. The procedure is primarily administered via endoscopy, enema, or oral delivery of freeze-dried preparations. The principal mechanisms of FMT include: **(A)** Alleviating gut microbial metabolic dysregulation in PD models; **(B)** reducing intestinal inflammation and barrier disruption; **(C)** mitigating blood–brain barrier impairment; **(D)** suppressing microglial and astrocyte activation in the nigrostriatal pathway; **(E)** inhibiting neuroinflammation; **(F)** downregulating TLR4/TNF-α signaling in gut-brain axis to protect dopaminergic neurons; **(G)** elevating striatal dopamine (DA) and serotonin (5-HT) levels.

Given the correlation between gut microbiota and PD, replacing disrupted microbial communities with healthy microbiota consortia may represent a potential therapeutic strategy (de Groot et al., 2017). Recent literature suggests that microbial intervention holds substantial potential for modulating microbiota dysbiosis-driven neurodegenerative disorders. Among these approaches, fecal microbiota transplantation (FMT) represents an emerging and safe therapeutic strategy (Figure 4).

FMT represents the most effective approach for reconstructing a patient’s overall gut microbiota. This technique involves transplanting functional microbial communities from healthy donor feces into the gastrointestinal tract of PD patients, thereby establishing a new gut microbiota ecosystem. The resulting enhancement of microbial diversity and functionality may contribute to effective PD treatment (Zhang et al., 2018). FMT is primarily administered via endoscopy, enema, or oral delivery of freeze-dried preparations. This therapeutic approach has now gained widespread recognition for its potential in treating neurological disorders. FMT may modulate the gut microbiota through neural, immune, and endocrine pathways, thereby influencing neurological symptoms. Currently, this technique is clinically applied in combination with antibiotics for treating Clostridioides difficile infection (Zhernakova et al., 2016; Boicean et al., 2023). FMT restores bacterial equilibrium in the gut, thereby reducing opportunities for pathogenic bacterial overgrowth. Previous studies have demonstrated that FMT significantly ameliorates gut microbial dysbiosis in PD mouse models, attenuates intestinal inflammation and barrier disruption, improves blood–brain barrier integrity, and reduces microglial and astrocytic activation in the nigrostriatal pathway. Furthermore, FMT suppresses neuroinflammation by downregulating TLR4/TNF-*α* signaling components in both gut and brain tissues, ultimately protecting dopaminergic neurons while increasing striatal dopamine (DA) and 5-HT levels (Sun et al., 2018; Zhang et al., 2021).

Currently, no single probiotic strain has been definitively proven to directly remodel dopaminergic neurons or ameliorate clinical manifestations of PD. However, emerging research has identified several key bacterial taxa that may exert neuroprotective effects through indirect mechanisms, representing promising candidates for microbiota-based PD intervention. We focus on four primary categories: SCFA (notably Butyrate), *Bacteroides fragilis*, Bifidobacterium, and Lactobacillus. Butyrate, as a pleiotropic metabolite, demonstrates potent anti-neuroinflammatory properties, enhances intestinal barrier integrity, and provides neuroprotection, thereby fostering a conducive microenvironment for dopaminergic neuron survival (Abreu et al., 2025). SCFAs derived from gut microbiota sustain host-microbial symbiosis by suppressing excessive immune responses and protecting commensal bacteria from elimination. Simultaneously, they enhance intestinal barrier stability and reduce permeability to prevent invasion by pathogenic microorganisms. Evidence indicates that SCFAs restrict neutrophil chemotaxis and monocyte–macrophage activity while promoting regulatory T cell (Treg) differentiation. Furthermore, SCFAs mitigate intestinal epithelial damage and modulate tight junction proteins to maintain barrier integrity (Wang et al., 2023). A 2024 randomized, double-blind, controlled clinical trial (Zeng et al., 2024) demonstrated that the combination of *Bacteroides fragilis* BF839 and earthworm protein effectively ameliorates both motor and non-motor symptoms in PD patients without significant adverse effects, offering a novel therapeutic approach. The underlying mechanism involves this formulation significantly increasing fecal Enterococcus abundance in PD patients, wherein Enterococcus has been shown to elevate striatal dopamine levels and improve behavioral phenotypes in PD mouse models. Bifidobacterium and Lactobacillus often function synergistically to produce neurotransmitters such as GABA and acetylcholine, while also facilitating the generation of L-tyrosine—a precursor to dopamine—thereby indirectly modulating neurological function (Yunes et al., 2016). Furthermore, Bifidobacterium alone exerts neuroprotective effects by preventing the reduction of spine density in Parkinson’s disease models. PD models treated with Bifidobacterium also demonstrate improved motor symptoms and reduced TNF-*α* levels in the striatum (Li et al., 2022).

### 3.2 FMT in animal models of PD

Recent studies demonstrate that FMT from healthy donors exhibits significant therapeutic effects in PD. Experimental investigations revealed that oral administration of 200 μL fecal bacterial suspension from healthy donors for 7 consecutive days induced the following physiological alterations in murine models: suppressed activity of the TLR4/TBK1/NF-κB/TNF-α signaling pathway in both gut and brain tissues; restored gut microbial homeostasis accompanied by decreased SCFAs levels; and reduced dopamine and 5-HT concentrations, ultimately leading to markedly attenuated glial cell activation. These findings provide novel insights for PD treatment strategies. Zhou et al. (2019) investigated an innovative dietary intervention strategy. Their study demonstrated that a fasting-mimicking diet (FMD) effectively inhibited the degeneration of dopaminergic neurons, thereby attenuating neurodegenerative progression. In the experimental paradigm, researchers transplanted gut microbiota from FMD-treated healthy mice into antibiotic-pretreated PD model mice, observing significantly elevated dopamine levels in the substantia nigra of recipient animals. This finding suggests that FMD may exert its neuroprotective effects through modulation of gut microbial communities. In 2021, Zhao et al. (2021) proposed the application of fecal microbiota transplantation (FMT) for therapeutic intervention in PD patients. Their experimental protocol involved daily FMT administration to 15 mice in the treatment group, with motor function assessments conducted at weeks 4 and 6 using standardized behavioral tests: rotarod test for motor coordination, adhesive removal test for sensorimotor integration, grip strength test for limb muscle strength, and pole test for movement balance. The results demonstrated significant improvement in motor symptoms in FMT-treated mice compared to rotenone-intoxicated controls. Furthermore, gastrointestinal function evaluations revealed that FMT treatment markedly increased fecal output in rotenone-induced PD mice. A recent Chinese study (Yang H. et al., 2024) administered fecal microbiota from PD patients to mice via oral gavage and systematically evaluated motor/intestinal functions, along with inflammatory and pathological changes in both colon and brain tissues. Fecal microbiota from PD patients exacerbated inflammation and neurodegeneration in A53T mice. This was evidenced by aggravated gut inflammation, impaired intestinal barrier function, enhanced microglial and astrocytic activation, aberrant *α*-synuclein deposition, and loss of dopaminergic neurons in the brain.

### 3.3 Clinical studies of FMT in PD treatment

Since 2017, levodopa-carbidopa intestinal gel (LCIG) delivered via percutaneous endoscopic gastrostomy (PEG) tubes has been clinically implemented for PD treatment (Bernier et al., 2017). This therapeutic approach involves continuous intestinal levodopa infusion through an enteral feeding system with intragastric placement. The standardized protocol commences with enteral nutrition initiation using a 15-Fr (5-mm) LCIG PEG-J tube via the gastric port, followed by nocturnal nutrition delivery through a feeding pump while temporarily suspending LCIG infusion. Over subsequent weeks, five daily bolus administrations are gradually introduced alongside continuous LCIG delivery. While particularly beneficial for advanced PD patients presenting with dysphagia, this treatment modality may require discontinuation due to complications including severe gastroparesis or new-onset aspiration pneumonia, ultimately necessitating surgical jejunostomy to establish alternative jejunal feeding access. In 2021, Freire-Alvarez et al. (2021) similarly demonstrated the efficacy of LCIG in managing motor complications in advanced PD patients. In 2018, another study (Fernandez et al., 2018) employed continuous levodopa administration via PEG-J delivery of LCIG, achieving stable plasma levodopa concentrations and reduced motor fluctuations in advanced PD cases. While this approach significantly decreased ‘off’ time in PD patients, 94% of participants nevertheless experienced device-related complications and adverse events. A 2023 report (Chernova et al., 2023) described a technical modification involving LCIG administration via PEG-J tubes for modulating gut microbiota in PD patients. LCIG infusion represents an effective symptomatic treatment for motor fluctuations refractory to conventional oral therapy. However, despite LCIG treatment, the patient exhibited progressive symptom deterioration characterized by persistent dyskinesias and off periods, accompanied by apathy, depression, and constipation. FMT has demonstrated significant efficacy in alleviating PD-associated constipation, characterized by an increased abundance of Firmicutes and decreased proportions of Proteobacteria and Bacteroidetes in treated patients. These microbial compositional changes correlate with effective amelioration of both constipation and tremor symptoms (Huang et al., 2019). Multiple studies comparing fecal samples between Parkinson’s disease patients and healthy controls have consistently revealed a characteristic pattern: PD patients typically exhibit a reduced Firmicutes/Bacteroidetes ratio. In 2015, Keshavarzian et al. (2015) conducted differential analysis of mucosal and fecal microbial communities in PD patients, definitively demonstrating a significant decrease in the Firmicutes/Bacteroidetes ratio. This dysbiotic signature was significantly correlated with α-syn misfolding and increased intestinal permeability, providing direct microbiological and pathological evidence supporting the “gut-origin” hypothesis of PD. A 2022 meta-analysis by Nishiwaki et al. (2022) systematically synthesized multiple studies and conclusively demonstrated that PD patients exhibit a consistent reduction in butyrate-producing Firmicutes alongside an increase in mucin-degrading Bacteroidetes within the gut microbiota. These findings directly support the mechanistic link between a decreased Firmicutes/Bacteroidetes ratio and both intestinal barrier disruption and exacerbated inflammation. Furthermore, this dysbiotic pattern was correlated with higher UPDRS-III scores, indicating more severe motor symptoms. In 2021, a study demonstrated that FMT significantly reduces Bacteroides abundance while increasing Prevotella and Blautia populations in PD patients with constipation. These microbial alterations were associated with marked improvements in both PAC-QOL (Patient Assessment of Constipation Quality of Life) and Wexner constipation scores, indicating substantial relief of constipation symptoms (Kuai et al., 2021). In 2021, Segal et al. (2021) conducted colonoscopic infusion of donor FMT in six PD patients, demonstrating significant improvements in motor symptoms, non-motor symptoms, and constipation over a six-month observation period. Two years later, DuPont et al. (2023) conducted a 12-week randomized, controlled, double-blind study involving 12 patients with mild-to-moderate PD. The study demonstrated that oral administration of freeze-dried FMT products effectively alleviated constipation and improved intestinal motility in PD patients by enhancing gut microbiome diversity. Several months later, Cheng et al. (2023) developed a novel fecal microbiota powder capsule formulation, which was subsequently validated in clinical trials to improve autonomic nervous system and gastrointestinal symptoms in PD patients. In 2024, Bruggeman et al. (2024) conducted a nasoduodenal FMT trial involving 43 early-stage PD patients. Post-transplantation assessments revealed significant improvements in both motor symptoms and constipation compared to baseline. Notably, the study also demonstrated FMT-mediated enhancement of cognitive functions, including amelioration of anxiety, depressive symptoms, and sleep disturbances. However, this investigation had several limitations. Specifically, since early-stage PD patients typically receive levodopa-based pharmacotherapy, it was methodologically challenging to completely eliminate confounding effects attributable to levodopa treatment in this cohort. Some researchers propose that the partial therapeutic effects of FMT on PD symptoms may be attributed to reduced abundance of tyrosine decarboxylase-producing bacteria in feces, consequently enhancing levodopa bioavailability (Beckers et al., 2024). A recent study (Yang X. et al., 2024) employed fucoidan-assisted FMT in a PD mouse model, demonstrating successful mitigation of both peripheral and central inflammation alongside amelioration of dopaminergic neuronal damage (Table 2 comprehensively summarizes animal studies and clinical trials investigating FMT for PD treatment).

**Table 2:** Animal experiments and clinical studies of fecal microbiota transplantation in the treatment of Parkinson’s disease.

Study Participants	FMT administration routes, formulations, and dosages	Duration	Mechanisms	Therapeutic efficacy of fecal microbiota transplantation (FMT)	References
MPTP-treated mice	Oral gavage Gavage administration of 200 μL bacterial suspension	7 Days	Restoration of depleted dopamine (DA) and serotonin (5-HT) levels	↓Gut microbiota dysbiosis ↓SCFA ↓Microglial and astrocytic activation ↓TLR4/TNF-α expression	Sun et al. (2018)
MPTP-treated mice	Oral gavage	2 Months	Inhibition of dopaminergic neuronal degeneration	Restoration of gut microbiota homeostasis ↓Gastrointestinal dysfunction and motor impairments ↓Blood–Brain barrier (BBB) Impairment ↓LPS, TLR4, MyD88, NF-κB	Zhou et al. (2019) and Zhao et al. (2021)
MPTP-treated mice	Oral gavage	1 Months	Restoration of gut microbiota homeostasis; Attenuation of Blood–Brain barrier (BBB) damage	↑Inflammation and neurodegeneration in A53T α-synuclein transgenic mice	Yang H. et al. (2024)
A 71-Year-old patient with Parkinson’s disease	Levodopa-carbidopa intestinal gel (LCIG) Percutaneous endoscopic gastrostomy with jejunal extension (PEG-J): Initiation of enteral nutrition through gastric port with nocturnal tube feeding, transitioning to bolus administration after 12 weeks	Several months	Restoration of gut microbiota homeostasis	Improvement of malnutrition secondary to dysphagia	Bernier et al. (2017)
262 Patients with Parkinson’s disease	Levodopa-carbidopa intestinal gel (LCIG) administration via percutaneous endoscopic gastrojejunostomy (PEG-J)	12 Weeks	Reduction of “Off” time and extension of dyskinesia-free “On” time	Reduction in “Off” time Alleviation of motor symptoms	Fernandez et al. (2018) and Freire-Alvarez et al. (2021)
A 49-year-old patient with Parkinson’s disease	Levodopa-carbidopa intestinal gel (LCIG) via percutaneous endoscopic gastrojejunal tube with direct infusion of fecal microbiota transplantation (FMT) complex (198 mL fecal suspension)	1 Year	Restoration of gut microbiota homeostasis Absence of potential interference with LCIG efficacy	Alleviation of motor symptoms	Chernova et al. (2023)
A 71-year-old patient with Parkinson’s disease	Endoscopic tube Delivered fecal microbiota suspension (200 mL) via transendoscopic enteral tube (TET): once daily for 3 consecutive days	1 Week	Enhancement of gut microbiota α-diversity	Daily regular bowel movements Resolution of lower limb tremor	Huang et al. (2019)
11 Patients with Parkinson’s disease	Nasoduodenal tube Infusion of 40-50 mL frozen fecal microbiota suspension	12 Weeks	Potentially associated with increased microbial abundance	Improvement in motor symptoms (Hoehn & Yahr stage and UPDRS scores) Reduction in PAC-QOL and Wexner constipation scores	Kuai et al. (2021)
6 Patients with Parkinson’s disease	Donor fecal microbiota transplantation (FMT) via colonoscopic delivery Three-part infusion of 300 mL donor fecal suspension	2 Years	Modulation of levodopa bioavailability	Improvement in motor, non-motor, and constipation symptoms	Segal et al. (2021)
12 Patients with Parkinson’s disease	Oral administration of lyophilized FMT preparations	12 Weeks	Activation of microglia and astrocytes; Reduction of gut microbiota alterations and inflammation	Improvement in constipation and gastrointestinal motility symptoms	DuPont et al. (2023)
56 Patients with Parkinson’s disease	Oral administration of lyophilized fecal microbiota powder capsules (prepared from 50 g donor stool)	10 Months	Improvement of gut microbiota α- and β-diversity	Improvement in autonomic symptoms (UPDRS part III scores and Group × Time effects) improvement in gastrointestinal symptoms	Cheng et al. (2023)
43 Patients with Parkinson’s disease	Autologous FMT via nasojejunal tube	1 Year	Alterations in gut microbiome following autologous transplantation	Improvement in motor, cognitive, and constipation symptoms	Bruggeman et al. (2024)

Current evaluations of FMT efficacy for PD symptom improvement across various administration routes primarily utilize the Hoehn-Yahr (H-Y) staging, Unified Parkinson’s Disease Rating Scale (UPDRS), and Non-Motor Symptoms Questionnaire (NMSS) to assess both motor and non-motor symptom improvements. However, existing clinical studies provide limited evidence to determine the optimal administration route. Experimental models have not yet established whether microbial-host interactions in the colon versus small intestine are more relevant to PD pathology, though different FMT routes may preferentially affect specific gastrointestinal regions. Given that most PD patients present with both motor and non-motor symptoms, effective management of these manifestations remains a clinical priority. Based on comparative analysis of FMT delivery methods, colorectal FMT infusion appears to be the most efficacious and durable approach for PD treatment. This method demonstrates comprehensive improvements in motor/non-motor symptoms and constipation relief in most patients. However, potential levodopa bioavailability alterations may exacerbate dyskinesias, necessitating further validation in larger cohorts (Segal et al., 2021). Additionally, while colorectal FMT infusion is generally safe, its invasive nature limits patient acceptability. For practicality, oral FMT capsules represent an emerging and more acceptable alternative, particularly for gastrointestinal symptom relief, though their efficacy for motor symptom improvement currently remains inferior to colorectal administration.

In summary, FMT demonstrates therapeutic potential in ameliorating intestinal dysfunction, modulating neuroinflammation, and regulating neurotransmitter activity, thereby conferring neuroprotective effects. Compared to conventional PD medications, FMT exhibits superior safety profiles and broader mechanisms of action. However, various factors may influence recipient outcomes. Notably, a recent study (D’Amato et al., 2020) revealed that FMT from aged donor mice impaired spatial learning and memory in young recipients, while transplantation from aged mouse models to germ-free (GF) mice reduced fecal short-chain fatty acid (SCFA) production, exacerbating depression-like behaviors and short-term memory deficits. Current research still lacks standardized donor screening protocols, and inadequate preparation may potentially yield countertherapeutic effects. The existing FMT technique involves transplanting fecal microbiota into recipient intestines, which reduces lipopolysaccharide (LPS) levels in both the colon and serum. This reduction subsequently inhibits the TLR4/MyD88/NF-κB signaling pathway and its downstream proinflammatory mediators in colonic tissues (Zhao et al., 2021). Furthermore, some researchers have proposed the application of Artificial Intelligence -guided imaging sensors for non-invasive monitoring and diagnosis of PD (Li et al., 2024). However, this technology remains in its nascent stages and requires further research for optimization and refinement.

Notably, several critical parameters—including optimal timing, dosage, donor characteristics, and patient selection criteria may serve as confounding factors influencing FMT efficacy. To optimize FMT’s therapeutic potential for PD treatment and achieve optimal clinical outcomes, ongoing research addressing these variables must be pursued (Donnelly et al., 2020). Furthermore, the efficacy of FMT is influenced not only by the gut microbiota composition of the donor, but also by that of the recipient. Additional factors, including the administration method and preservation techniques of FMT preparations, also significantly contribute to treatment outcomes (Ng S. C. et al., 2020). Furthermore, the therapeutic efficacy of FMT varies substantially across different administration routes when treating conditions such as recurrent Clostridioides difficile infection, inflammatory bowel disease (IBD), diarrhea, and constipation (McGowan et al., 2020). A 2020 domestic clinical study (Xue et al., 2020) conducted a self-controlled trial involving 15 PD patients receiving FMT, with 10 patients administered FMT via the colonic route and 5 via the nasoduodenal route. The results demonstrated that colonoscopic FMT significantly improved various clinical assessment scores in PD patients, whereas nasoduodenal administration showed suboptimal therapeutic efficacy. Consequently, modern clinical practice preferentially employs the colonic route for FMT in PD treatment to achieve satisfactory therapeutic outcomes. Although FMT has emerged as an innovative therapeutic approach for PD, current clinical evidence remains insufficient. Comprehensive investigations are still required to address several critical aspects, including donor selection criteria, standardized processing of transplant specimens, duration of therapeutic effects, and optimal intervention timing. These efforts aim to minimize potential safety concerns associated with FMT, particularly during the COVID-19 pandemic (Wu et al., 2020). Furthermore, a study investigating dietary interventions and FMT (Kennedy et al., 2025) revealed that fecal microbiota transplantation exhibits limited efficacy when administered in suboptimal dietary contexts. These findings suggest that optimal therapeutic outcomes may require the synergistic combination of FMT with appropriate dietary modifications, presenting a significant challenge for future research in this field. Moreover, distinct patterns of gut microbiota dysbiosis have been identified across different PD subtypes, necessitating systematic investigations to validate the feasibility and efficacy of personalized FMT protocols tailored to individual patient profiles.

## 4 Conclusions and future perspectives

This review examines the potential mechanisms linking gut microbiota and the MGBA to PD pathogenesis, aiming to provide novel insights for future PD prevention and treatment through microbial modulation. While multiple studies have investigated alterations in fecal microbial abundance and clinical correlations in PD patients, substantial discrepancies persist due to variations in patient/control selection criteria and methodological approaches across studies.

Multiple factors can modulate the MGBA, among which FMT represents a promising therapeutic strategy for PD by directly targeting gut microbial composition. Future research in this field should focus on two key directions: Elucidating the precise mechanisms underlying gut microbial interventions and their association with PD pathogenesis through additional clinical studies; Investigating the effects of dietary modifications, physical exercise, and lifestyle adjustments on gut microbiota in PD patients. Future research on FMT for PD should address current challenges by adopting a precision medicine approach. This entails matching PD patients with optimal donors based on their microbial enterotypes to enhance treatment specificity. Furthermore, to overcome the limited efficacy of single-dose FMT, multi-course regimens or periodic maintenance therapies warrant investigation. Finally, exploring FMT-based combination therapies represents a promising frontier. The observed synergistic effects between FMT and Deep Brain Stimulation (DBS) in clinical trials provide a rationale for developing integrated strategies that combine FMT with PD-targeted medications or probiotic formulations. This “gut microbiota modulation with pathological target blockade” dual-intervention framework holds potential for superior therapeutic outcomes.
